# Supplementary material for: Associations between long-term drought and diarrhea among children under five in low- and middle-income countries
Source: Nat Commun. 2022 Jun 30;13:3661. doi: 10.1038/s41467-022-31291-7 (PMC9247069; doi:10.1038/s41467-022-31291-7)
Supplement: Supplementary file 1 — Supplementary information [file 41467_2022_31291_MOESM1_ESM.pdf]

Associations between long-term drought and diarrhea among children under five in low- and middle-income countries

Supplementary information

Pin Wang[1,2], Ernest Asare[3], Virginia E. Pitzer[3], Robert Dubrow[1,2], Kai Chen[1,2]

[1] Department of Environmental Health Sciences, Yale School of Public Health, New Haven, CT, USA

[2] Yale Center on Climate Change and Health, Yale School of Public Health, New Haven, CT, USA

[3] Department of Epidemiology of Microbial Diseases and the Public Health Modeling Unit, Yale School of Public Health, New Haven, CT, USA

Supplementary Table 1. Descriptive statistics for the number of drought-months experienced by included children during 1990–2019 by climate zone, drought severity, and drought timescale.

	Mean (SD)	Minimum	Median	Maximum	Interquartile range
**Tropical zone**					
Mild drought					
6-month	84.8 (9.7)	45	84	152	78–91
12-month	84.8 (13.2)	39	85	152	76–94
18-month	84.8 (14.2)	37	84	174	75–94
24-month	85.4 (17.1)	29	85	182	74–97
Severe drought					
6-month	37.3 (5.2)	11	38	57	34–41
12-month	37.8 (7.1)	4	38	64	34–42
18-month	37.5 (7.4)	4	38	68	33–42
24-month	37.2 (8.0)	6	37	67	32–42
**Temperate zone**					
Mild drought					
6-month	81.0 (8.7)	50	81	115	75–87
12-month	81.9 (13.4)	39	82	131	73–91
18-month	80.3 (13.0)	41	80	135	71–89
24-month	78.9 (15.2)	26	78	145	68–89
Severe drought					
6-month	37.0 (5.5)	18	37	57	34–41
12-month	36.1 (9.7)	2	37	63	31–43
18-month	35.8 (9.0)	2	37	63	31–42
24-month	36.1 (8.9)	5	37	67	31–42
**Dry zone**					
Mild drought					
6-month	83.2 (8.5)	53	84	112	78–89
12-month	85.6 (14.1)	33	86	132	76–96
18-month	83.9 (11.7)	37	84	128	76–92
24-month	82.5 (14.2)	26	83	130	73–92
Severe drought					
6-month	36.3 (6.5)	12	37	54	33–41
12-month	35.5 (9.6)	2	37	63	31–42
18-month	35.1 (8.8)	3	36	59	31–41
24-month	35.3 (8.8)	3	36	65	31–41

Note: 47,928, 26,818, and 33,143 missing values in the tropical, temperate, and dry zone, respectively, were not included.

Supplementary Table 2. Percentage of children under age five who had diarrhea in the two weeks preceding the interview in 43 countries during 1990-2019 in the dataset without missing values for baseline variables (*N*=713,918).

	Diarrhea incidence (%)	Number of children
Total	12·8	713,918
Age		
< 6 months	10·3	72,124
6–11 months	20·9	76,641
12–23 months	19·9	145,773
24–35 months	13·1	140,138
36–47 months	8·5	142,331
48–59 months	6·1	136,911
Sex		
Male	13·2	364,750
Female	12·4	349,168
Residence		
Urban	12·5	204,701
Rural	12·9	509,217
Mother's education		
No education	13·0	259,491
Primary	14·8	192,503
Secondary	11·5	219,456
Higher	9·1	42,468
Wealth quintile		
Lowest	15·5	117,827
Second	14·5	140,224
Middle	12·5	154,452
Fourth	11·6	161,550
Highest	10·4	139,865

Supplementary Table 3. Associations between diarrhea in children and drought at different timescales in different models.

	Crude model A	Crude model B	Main model
Sample size	1,305,061	713,918	713,918
Mild drought			
6-month	**1·05 (1·04-1·07)**	**1·07 (1·05-1·09)**	**1·05 (1·03-1·07)**
12-month	**1·03 (1·01-1·04)**	**1·07 (1·05-1·09)**	**1·06 (1·04-1·08)**
18-month	**1·02 (1·01-1·04)**	**1·07 (1·05-1·09)**	**1·06 (1·04-1·08)**
24-month	**1·05 (1·03-1·06)**	**1·08 (1·05-1·10)**	**1·08 (1·06-1·10)**
Severe drought			
6-month	**1·09 (1·07-1·11)**	**1·11 (1·08-1·14)**	**1·08 (1·05-1·11)**
12-month	1·01 (0·99-1·04)	1·03 (0·99-1·06)	1·03 (1·00-1·06)
18-month	1·01 (0·99-1·04)	1·01 (0·97-1·04)	1·03 (0·99-1·06)
24-month	1·01 (0·99-1·04)	1·00 (0·97-1·04)	1·03 (1·00-1·06)

Note: Significant results are marked in bold. Both crude models included the drought indicator, temperature, precipitation, and survey month and year. Main models additionally included age, sex, mother's education, urban/rural area of residence, and wealth index. Crude model A included all children in 141 surveys. Crude model B only included children with no missing values for all variables included in the main model.

Supplementary Table 4. Mediating effects of individual WASH variables (assessed in separate models) using the 4-way decomposition method allowing for interaction between 6-month drought and the WASH variable.

	Water source	Time to collect water	Toilet facility	Place to wash hands	Water availability	Soap availability	Water treatment
Mild drought							
Total excess RR (95% CI)	0.055	0.062	0.062	0.108	0.144	0.167	0.097
	(0.049–0.061)	(0.056–0.067)	(0.056–0.068)	(0.103–0.112)	(0.139–0.149)	(0.164–0.172)	(0.090–0.101)
CDE (95% CI)	0.013	0.047	0.008	0.057	0.061	0.067	0.086
	(-0.001–0.022)	(0.042–0.053)	(0.002–0.019)	(0.049–0.065)	(0.055–0.066)	(0.043–0.074)	(0.082–0.091)
INT_{ref} (95% CI)	0.040	0.008	0.049	0.022	0.056	0.086	0.011
	(0.037–0.047)	(0.008–0.009)	(0.043–0.053)	(0.020–0.025)	(0.049–0.064)	(0.077–0.105)	(0.002–0.017)
INT_{med} (95% CI)	0.001	0002	0.001	0.006	0.012	0.008	0.000
	(0.001–0.001)	(0.001–0.002)	(0.001–0.002)	(0.005–0.006)	(0.011–0.014)	(0.007–0.010)	(0.000–0.000)
PIE (95% CI)	0.001	0.004	0.004	0.023	0.014	0.006	0.000
	(0.001–0.001)	(0.004–0.005)	(0.004–0.004)	(0.022–0.024)	(0.012–0.015)	(0.005–0.006)	(0.000–0.001)
CDE proportion	23.3%	76.7%	13.0%	53.0%	42.7%	40.1%	88.4%
INT_{ref} proportion	72.6%	13.5%	78.8%	20.1%	38.9%	51.4%	10.9%
INT_{med} proportion	2.0%	2.6%	2.4%	5.2%	8.6%	5.0%	0.2%
PIE proportion	2.0%	7.2%	5.9%	21.8%	9.8%	3.6%	0.5%
Severe drought							
Total excess RR (95% CI)	0.116	0.131	0.131	0.236	0.340	0.383	0.204
	(0.104–0.129)	(0.119–0.143)	(0.118–0.143)	(0.227–0.244)	(0.323–0.354)	(0.374–0.395)	(0.188–0.213)
CDE (95% CI)	0.026	0.097	0.016	0.118	0.127	0.139	0.180
	(-0.002–0.044)	(0.085–0.108)	(0.003–0.039)	(0.101–0.134)	(0.114–0.136)	(0.087–0.154)	(0.170–0.190)
INT_{ref} (95% CI)	0.083	0.018	0.102	0.046	0.127	0.195	0.023
	(0.078–0.098)	(0.016–0.020)	(0.092–0.111)	(0.043–0.052)	(0.109–0.145)	(0.175–0.239)	(0.005–0.038)
INT_{med} (95% CI)	0.005	0.007	0.006	0.024	0.058	0.037	0.001
	(0.004–0.006)	(0.006–0.008)	(0.005–0.007)	(0.022–0.027)	(0.050–0.066)	(0.033–0.045)	(0.000–0.002)
PIE (95% CI)	0.002	0.010	0.007	0.048	0.029	0.012	0.001
	(0.002–0.002)	(0.009–0.010)	(0.007–0.008)	(0.045–0.050)	(0.025–0.032)	(0.010–0.012)	(0.001–0.001)
CDE proportion	22.3%	73.6%	12.4%	49.8%	37.3%	36.3%	87.8%
INT_{ref} proportion	71.9%	13.5%	77.6%	19.6%	37.2%	51.0%	11.3%
INT_{med} proportion	3.9%	5.6%	4.6%	10.3%	17.0%	9.7%	0.5%
PIE proportion	1.9%	7.3%	5.5%	20.3%	8.6%	3.0%	0.5%

WASH: water, sanitation, and hygiene; RR: relative risk; CI: confidence interval; CDE: controlled direct effect; INT_{ref}: reference interaction; INT_{med}: mediated interaction; PIE: pure indirect effect/mediated main effect. These mediating effect estimates were calculated by a 4-way decomposition method[1], in which the overall effect of drought can be decomposed into the proportion of the effect due to 1) the direct effect of drought (CDE); 2) only interaction between drought and WASH (INT_{ref}); 3) both mediation by WASH and the aforementioned interaction (INT_{med}); and 4) only mediation by WASH (PIE). INT_{ref} + INT_{med} means the overall effect attributable to interaction; INT_{med} + PIE means the overall effect mediated. Of note, this method does not allow non-linear covariates, random effects, or multiple mediators. Thus, mean temperature, rainfall, and survey year were included as linear terms, the random effect variable for survey sites was removed, and the mediating effect of each WASH variable was assessed in a separate model.

Supplementary Figure 1. Number of months with 6-month drought of different severity in 51 countries during 1990-2019.

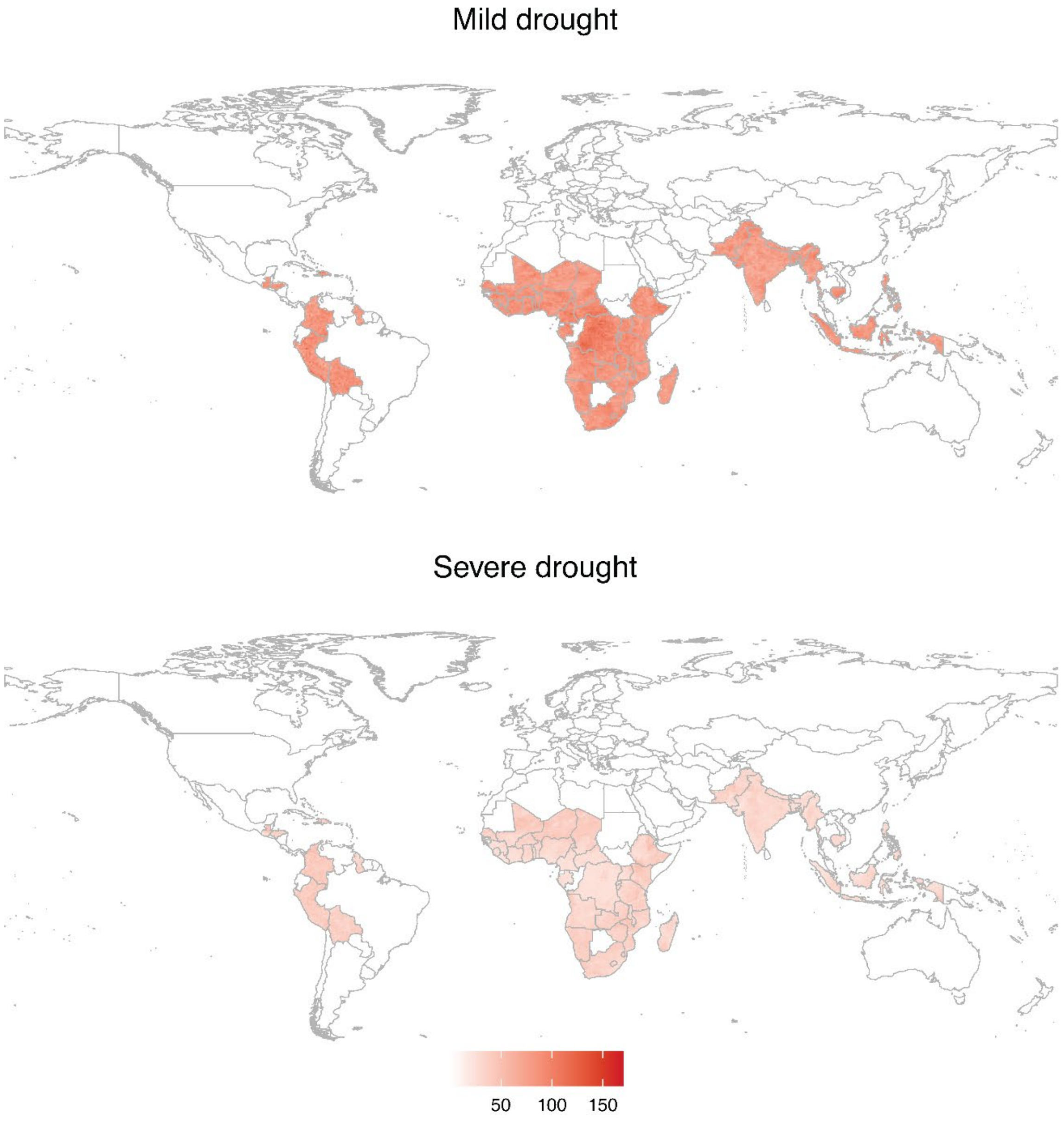

Supplementary Figure 2. Number of months with 12-month drought of different severity in 51 countries during 1990-2019.

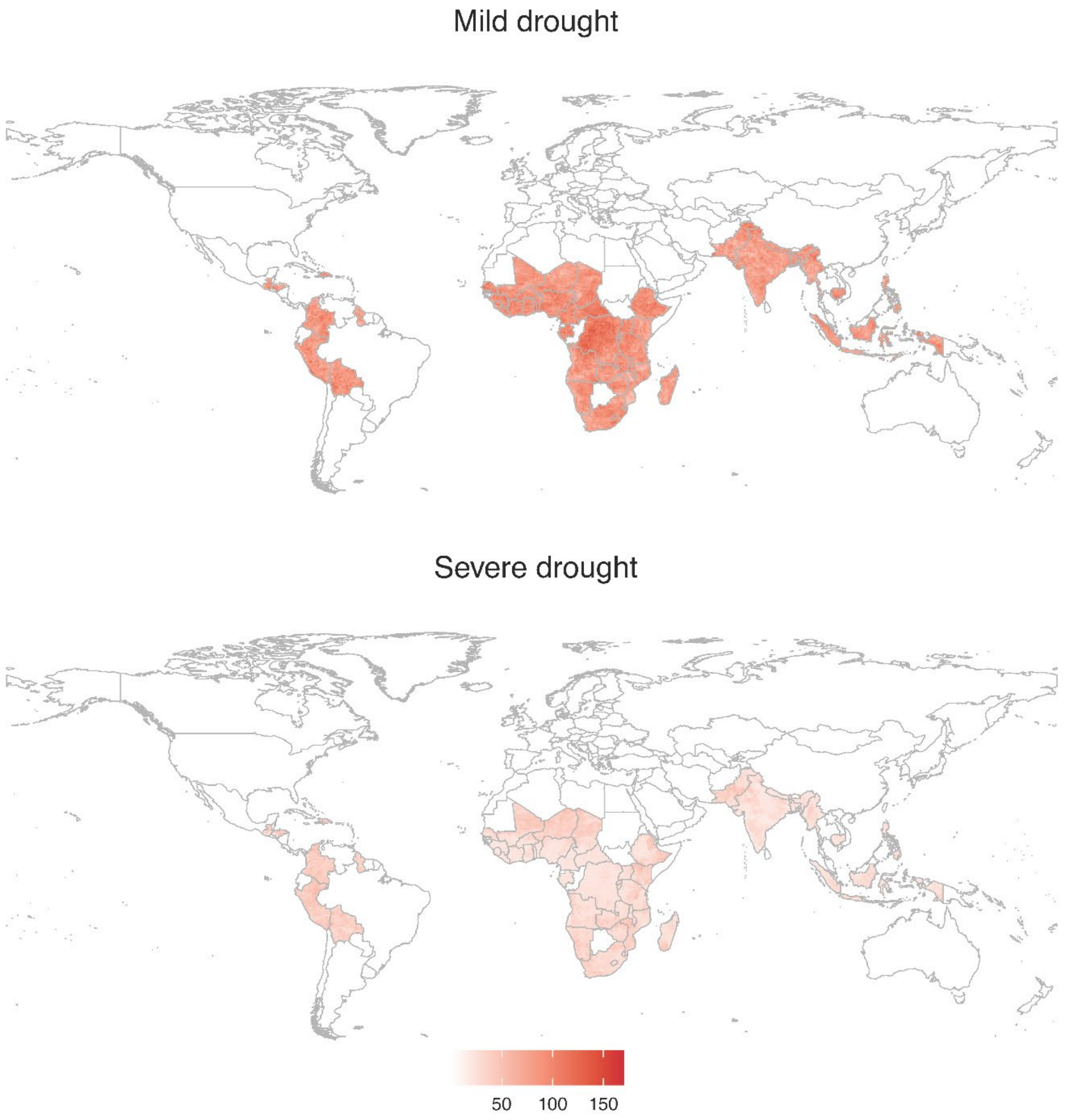

Supplementary Figure 3. Number of months with 18-month drought of different severity in 51 countries during 1990-2019.

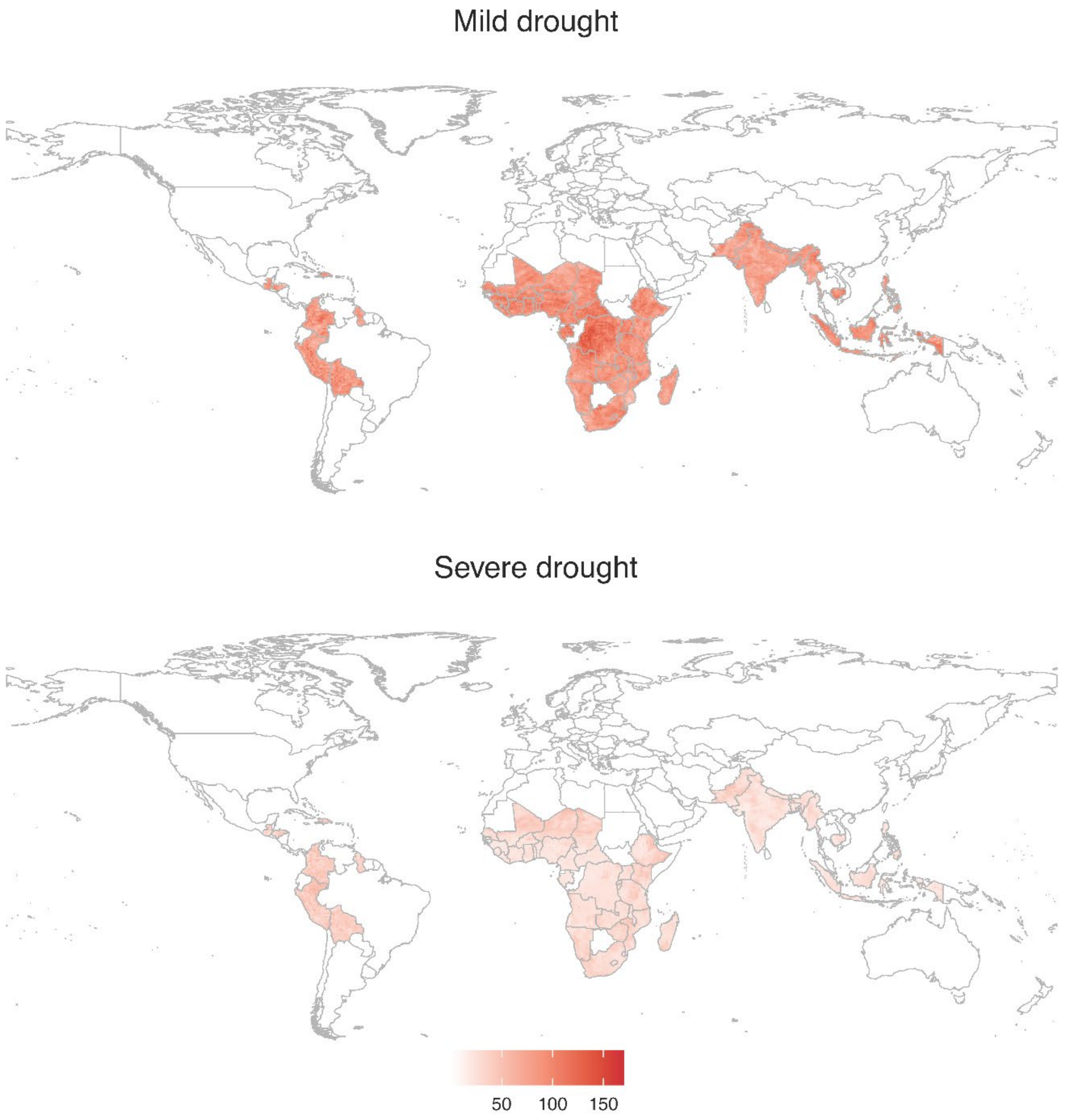

Supplementary Figure 4. Number of months with 24-month drought of different severity in 51 countries during 1990-2019.

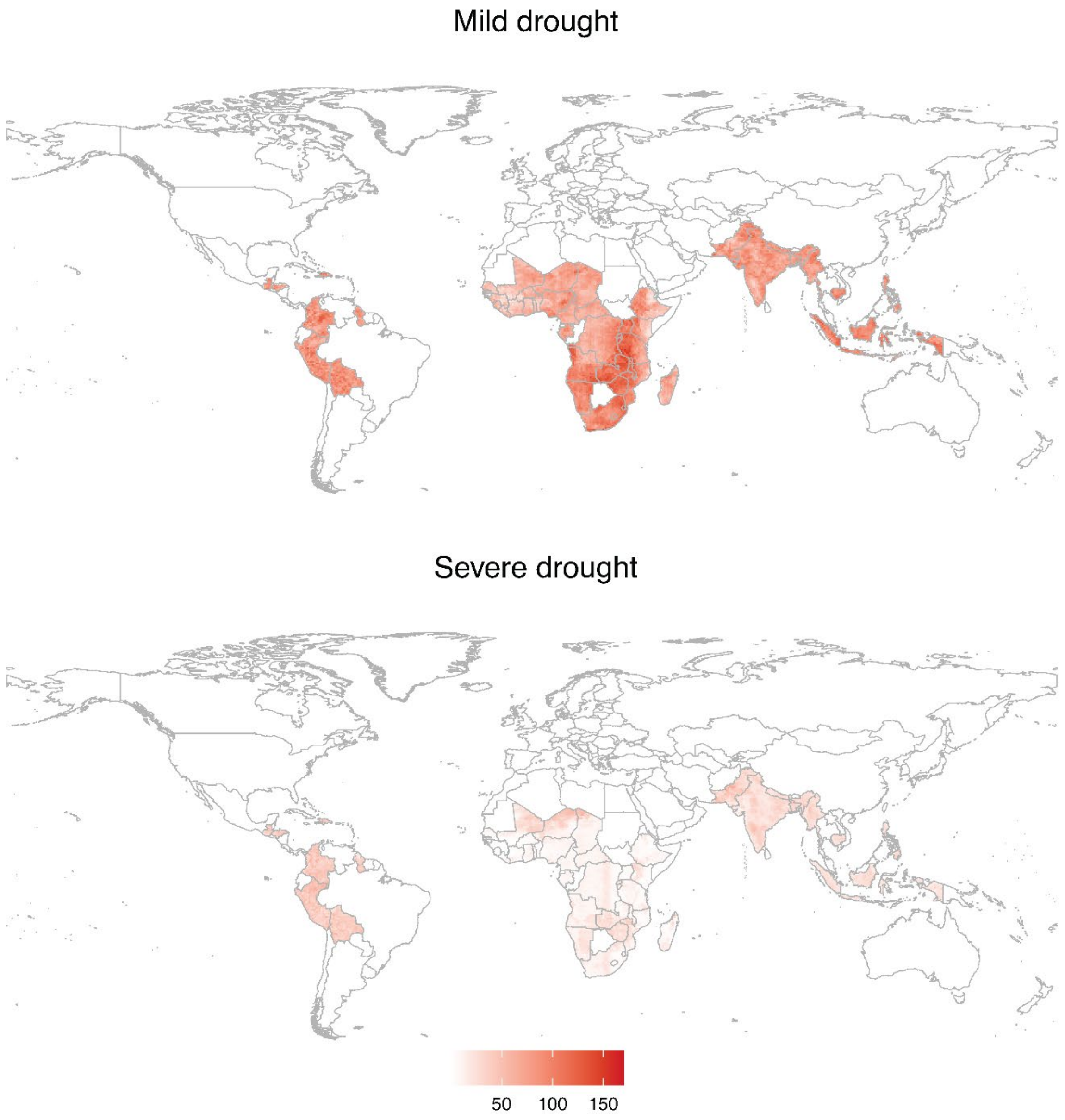

Supplementary Figure 5. Associations between diarrhea in children and drought at different timescales stratified by age group (N=713,956). Statistically significant pairwise differences are marked with an asterisk. Generalized linear mixed effect models were used and no adjustments were made for multiple comparisons. Data are presented as mean values +/- 1.96 × standard error. The p-values are two-sided.

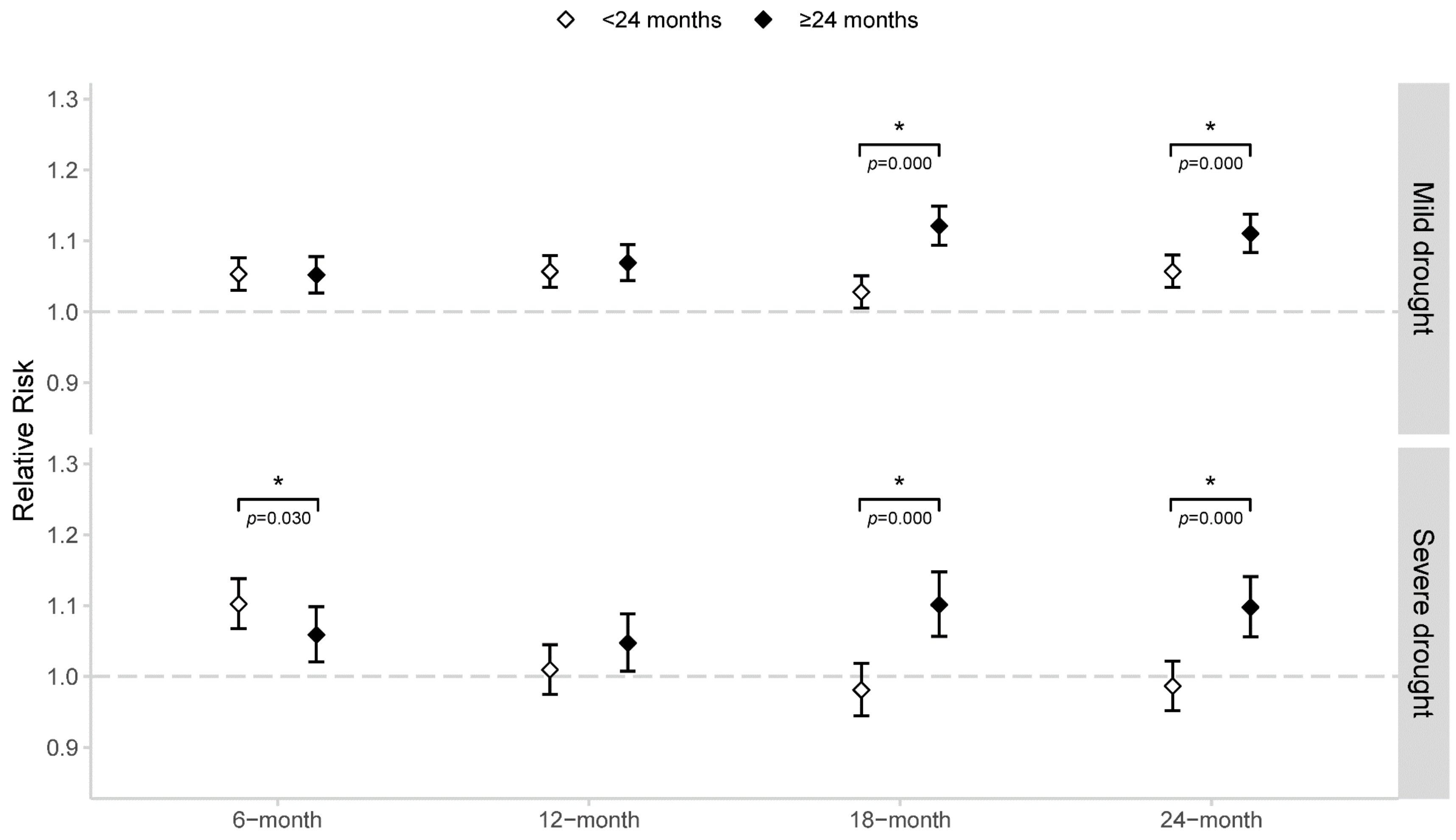

Supplementary Figure 6. Associations between diarrhea in children and drought at different timescales stratified by sex (*N*=713,956). Statistically significant pairwise differences are marked with an asterisk. Generalized linear mixed effect models were used and no adjustments were made for multiple comparisons. Data are presented as mean values +/- 1.96 × standard error. The p-values are two-sided.

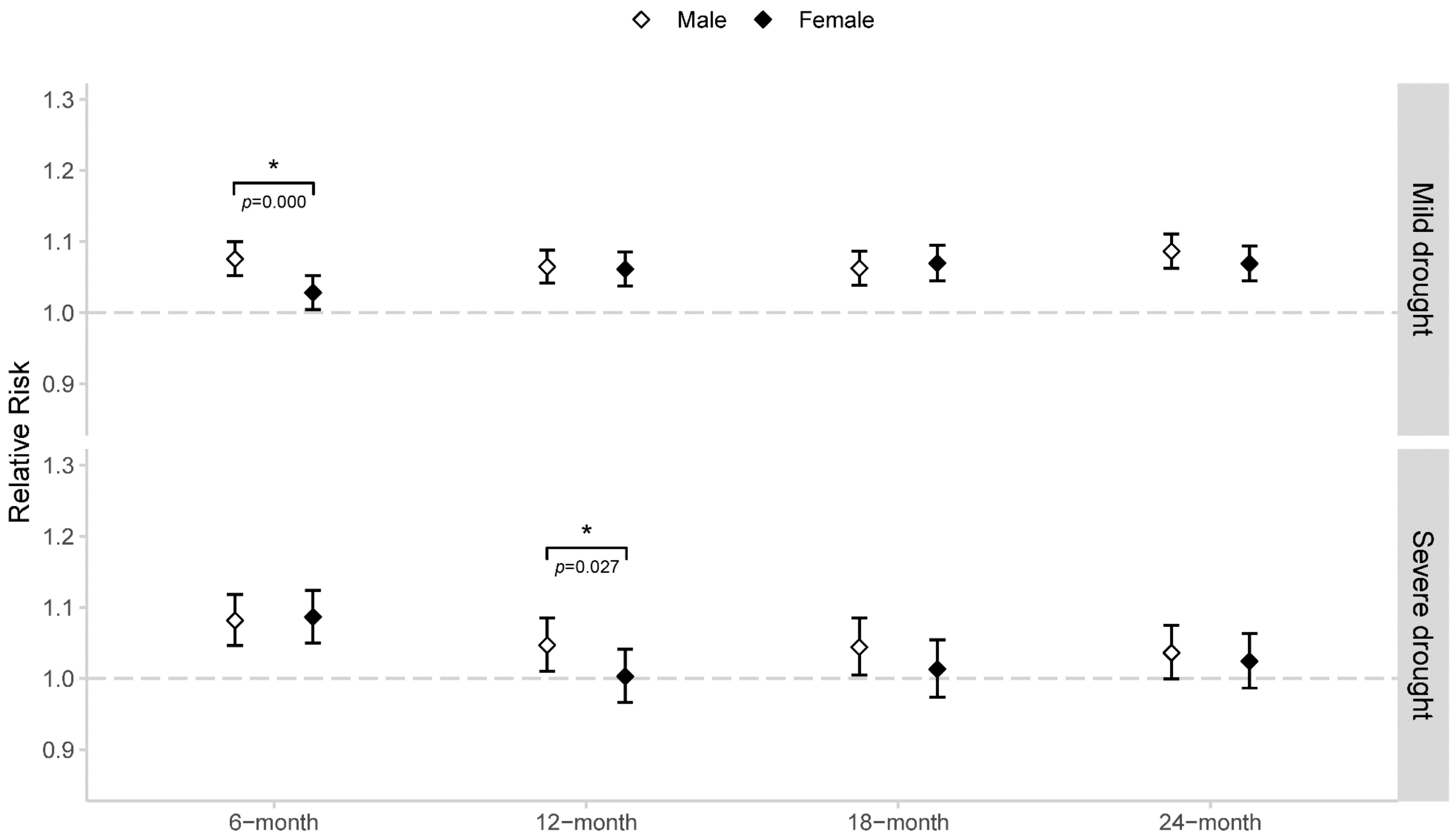

Supplementary Figure 7. Associations between diarrhea in children and drought at different timescales stratified by mother's education (N=713,956). Statistically significant pairwise differences are marked with an asterisk. Generalized linear mixed effect models were used and no adjustments were made for multiple comparisons. Data are presented as mean values +/- 1.96 × standard error. The p-values are two-sided.

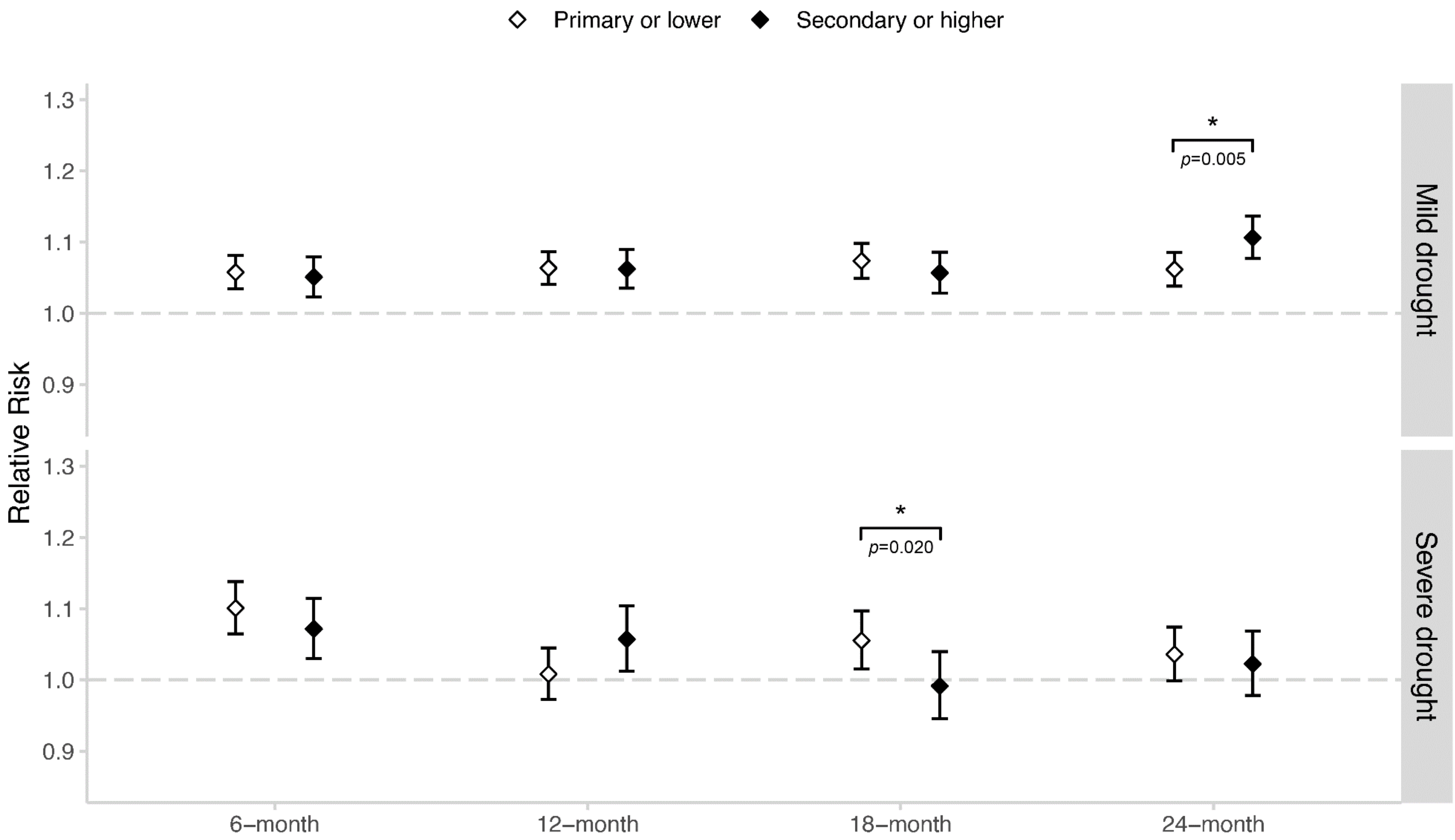

Supplementary Figure 8. Associations between diarrhea in children and drought at different timescales stratified by area of residence (*N*=713,956). Statistically significant pairwise differences are marked with an asterisk. Generalized linear mixed effect models were used and no adjustments were made for multiple comparisons. Data are presented as mean values +/- 1.96 × standard error. The p-values are two-sided.

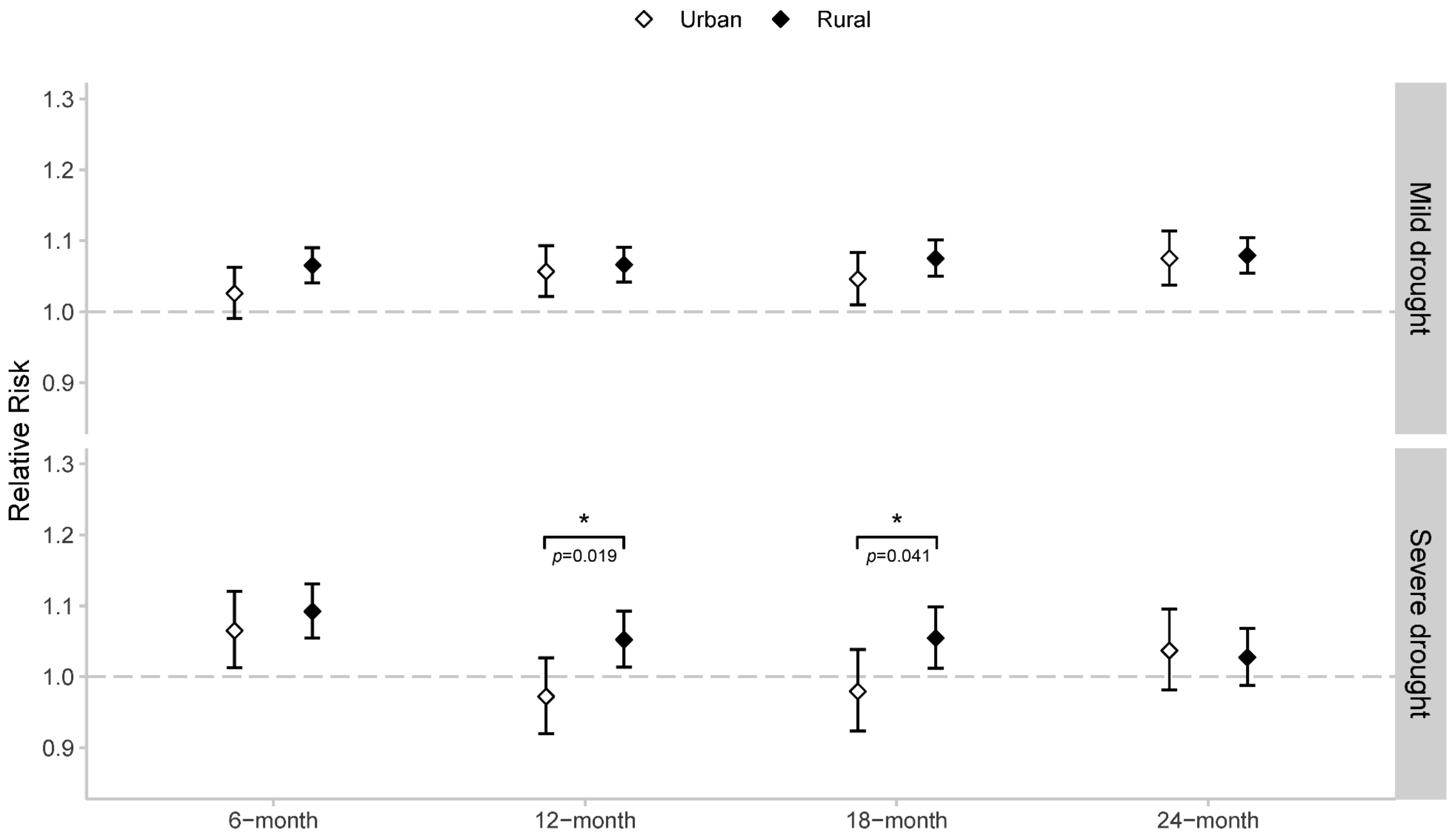

Supplementary Figure 9. Associations between diarrhea in children and drought at different timescales stratified by wealth index (N=713,956; lower: 1st–3rd quintiles, higher: 4th–5th quintiles). Statistically significant pairwise differences are marked with an asterisk. Generalized linear mixed effect models were used and no adjustments were made for multiple comparisons. Data are presented as mean values +/- 1.96 × standard error. The p-values are two-sided.

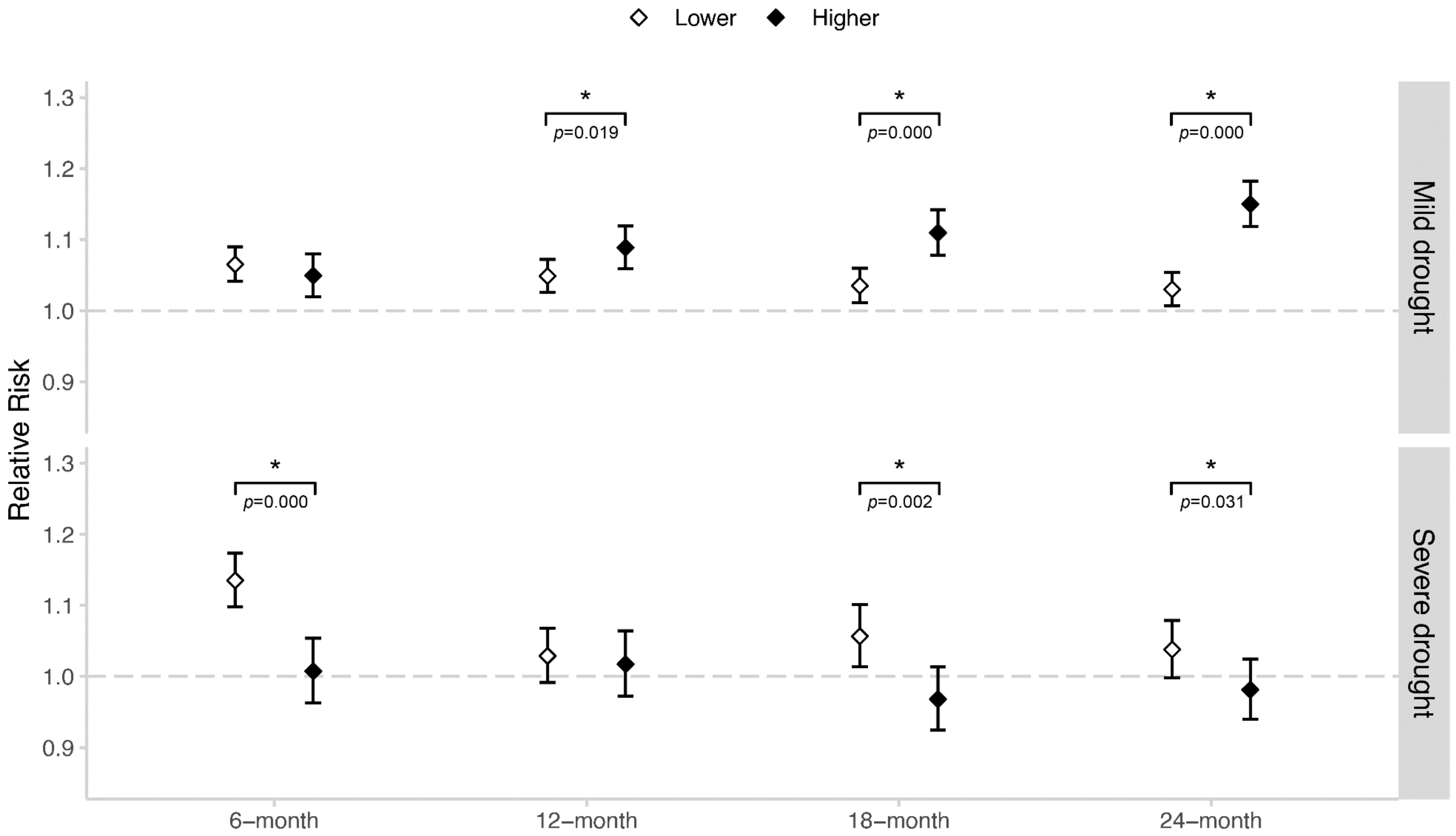

Supplementary Figure 10. Associations between diarrhea in children and drought at different timescales stratified by history of breastfeeding (*N*=471,310). Statistically significant pairwise differences are marked with an asterisk. Generalized linear mixed effect models were used and no adjustments were made for multiple comparisons. Data are presented as mean values +/- 1.96 × standard error. The p-values are two-sided.

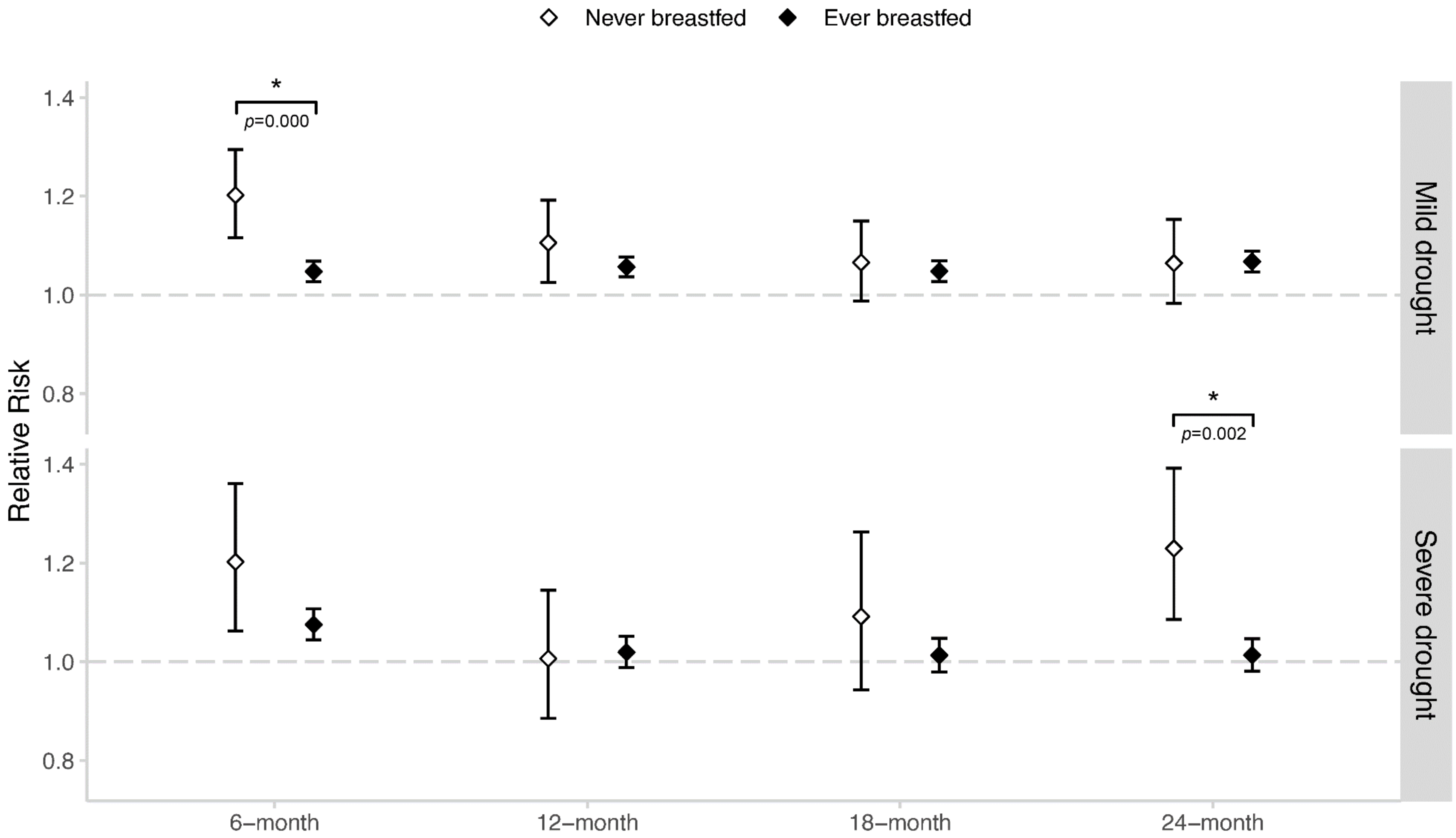

Supplementary Figure 11. Associations between diarrhea in children and drought at different timescales stratified by source of drinking water (*N*=708,711). Statistically significant pairwise differences are marked with an asterisk. Generalized linear mixed effect models were used and no adjustments were made for multiple comparisons. Data are presented as mean values +/- 1.96 × standard error. The p-values are two-sided.

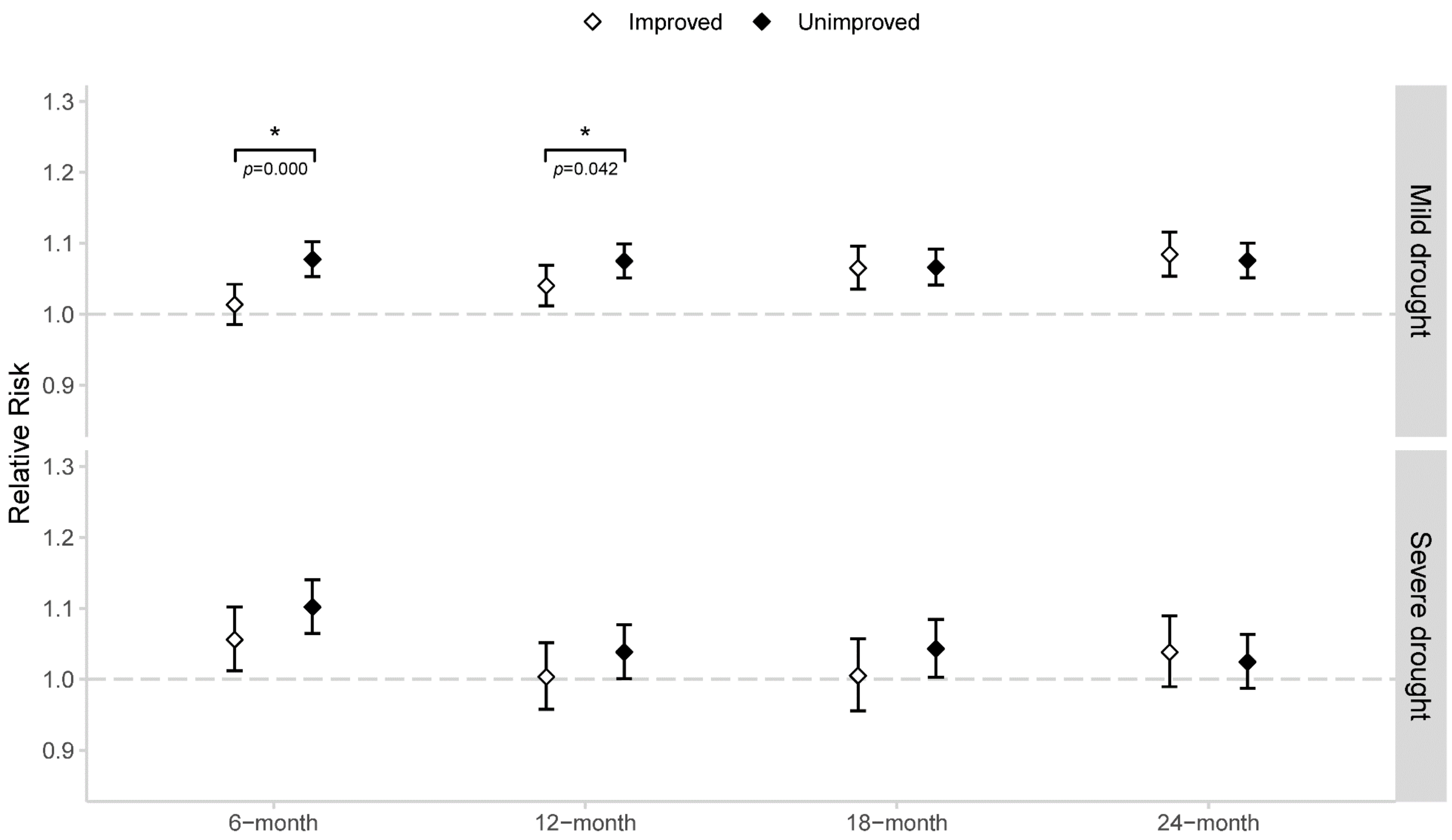

Supplementary Figure 12. Associations between diarrhea in children and drought at different timescales stratified by drinking water treatment (N=683,119). Statistically significant pairwise differences are marked with an asterisk. Generalized linear mixed effect models were used and no adjustments were made for multiple comparisons. Data are presented as mean values +/- 1.96 × standard error. The p-values are two-sided.

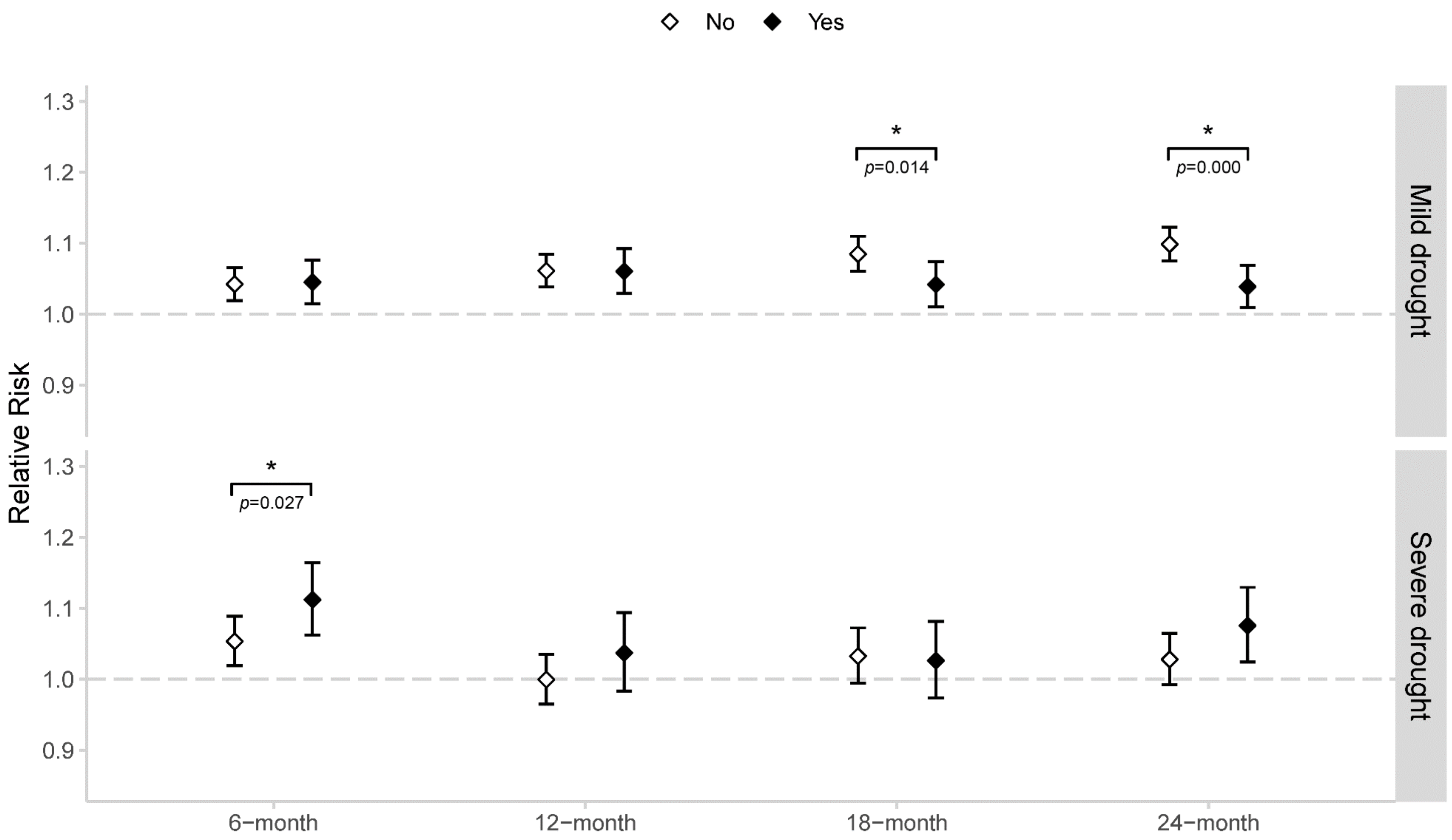

Supplementary Figure 13. Associations between diarrhea in children and drought at different timescales stratified by place to wash hands (*N*=553,843). Statistically significant pairwise differences are marked with an asterisk. Generalized linear mixed effect models were used and no adjustments were made for multiple comparisons. Data are presented as mean values +/- 1.96 × standard error. The p-values are two-sided.

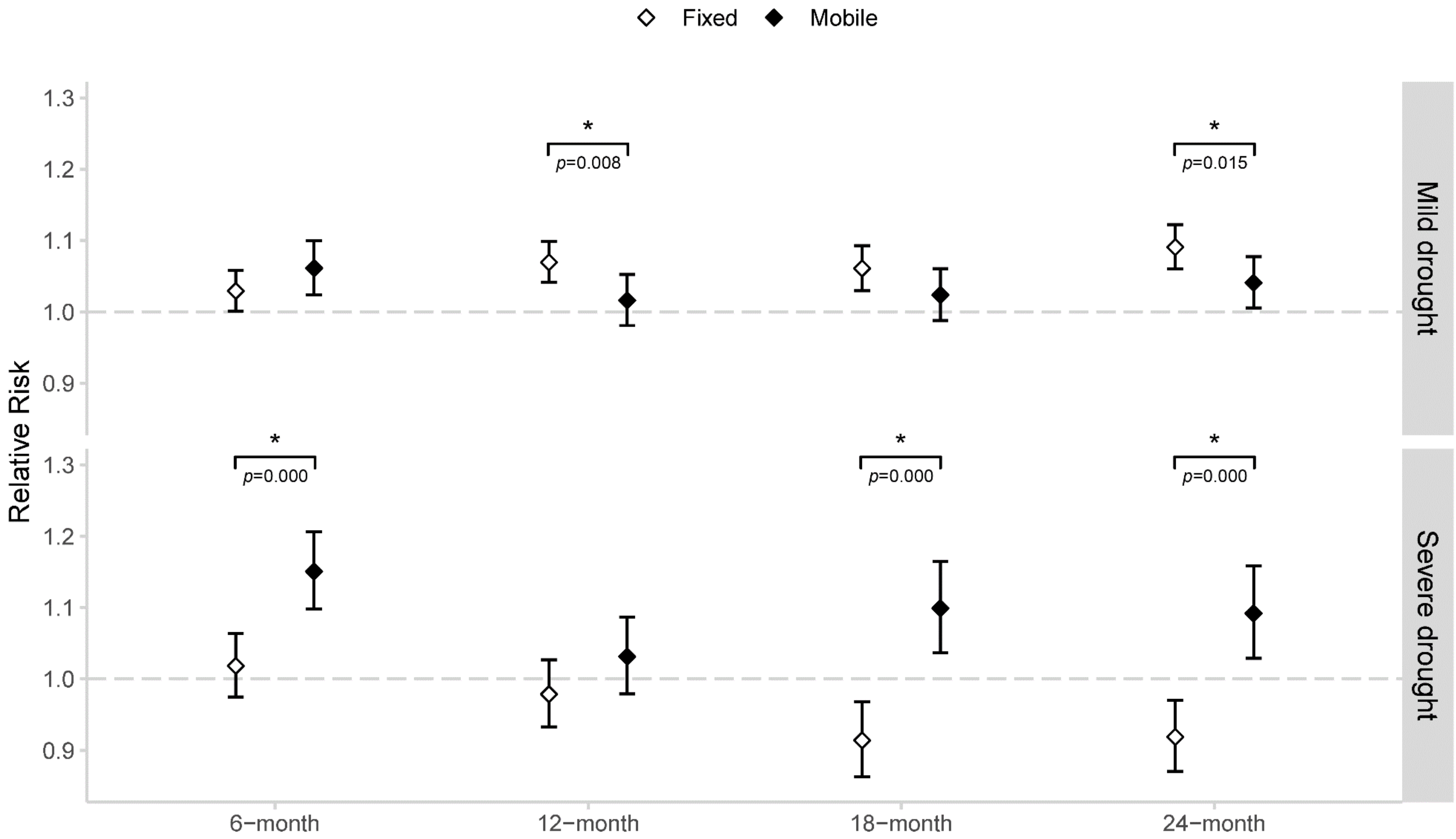

Supplementary Figure 14. Associations between diarrhea in children and drought at different timescales stratified by toilet facility (N=702,386). Statistically significant pairwise differences are marked with an asterisk. Generalized linear mixed effect models were used and no adjustments were made for multiple comparisons. Data are presented as mean values +/- 1.96 × standard error. The p-values are two-sided.

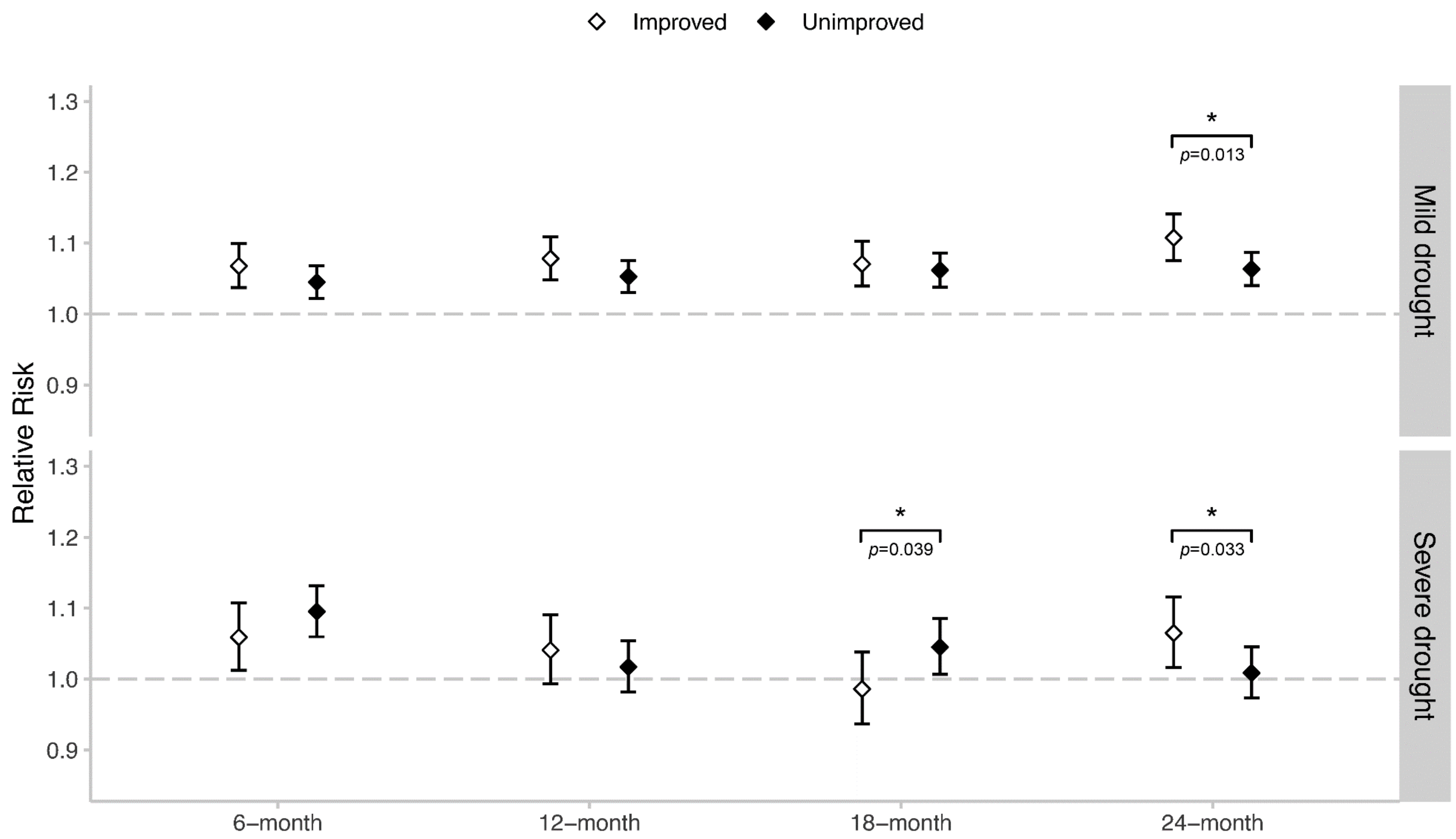

Supplementary Figure 15. Associations between diarrhea in children and drought at different timescales using the SPEI from the Global SPEI database (*N*=704,040; SPEI: standardized precipitation evapotranspiration index). Generalized linear mixed effect models were used and no adjustments were made for multiple comparisons. Data are presented as mean values +/- 1.96 × standard error.

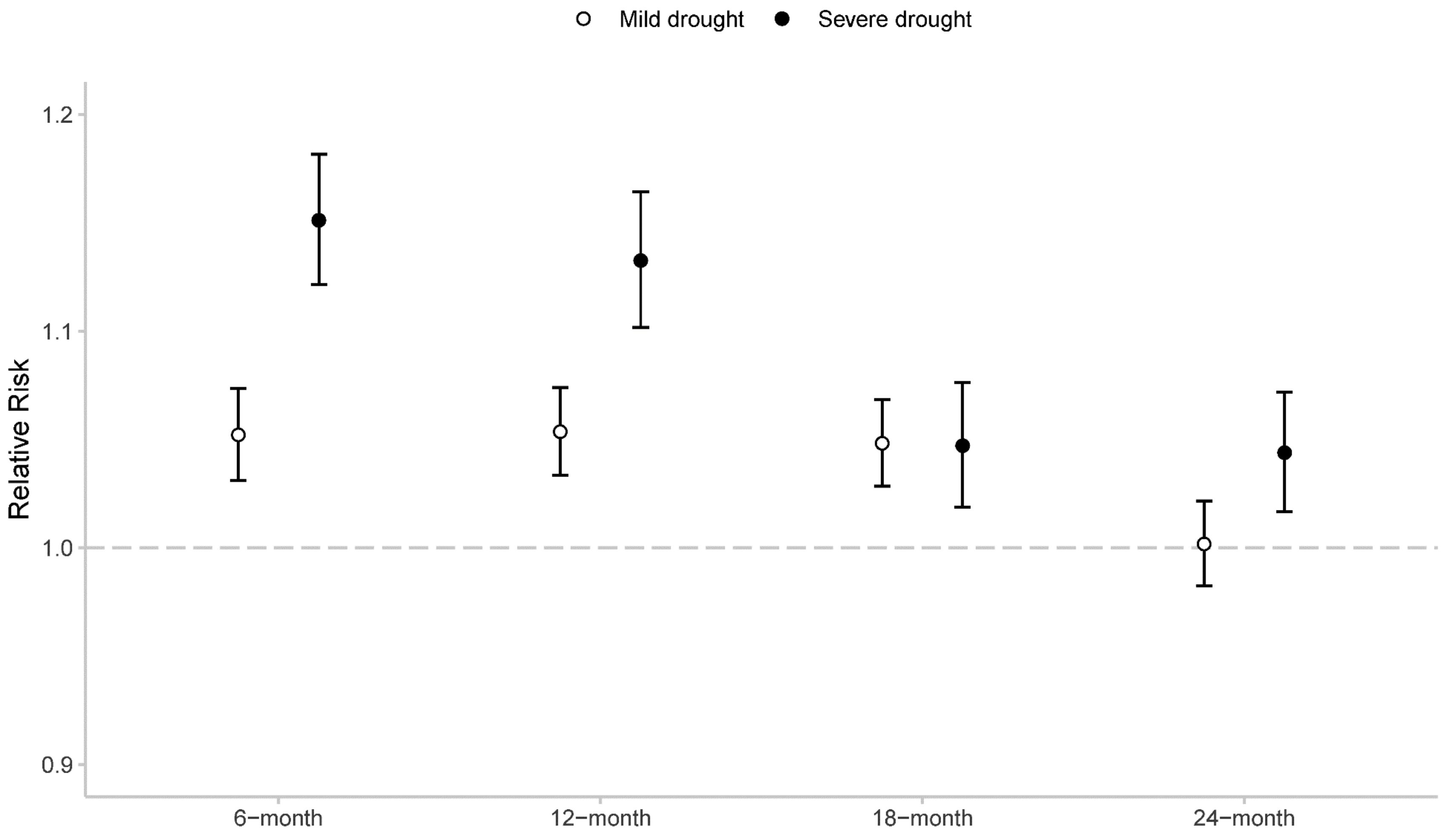

Supplementary Figure 16. Associations between diarrhea in children and drought at different timescales using the SPEI from the European Space Agency (only timescales consistent with this study were used; *N*=713,750; SPEI: standardized precipitation evapotranspiration index). Generalized linear mixed effect models were used and no adjustments were made for multiple comparisons. Data are presented as mean values +/- 1.96 × standard error.

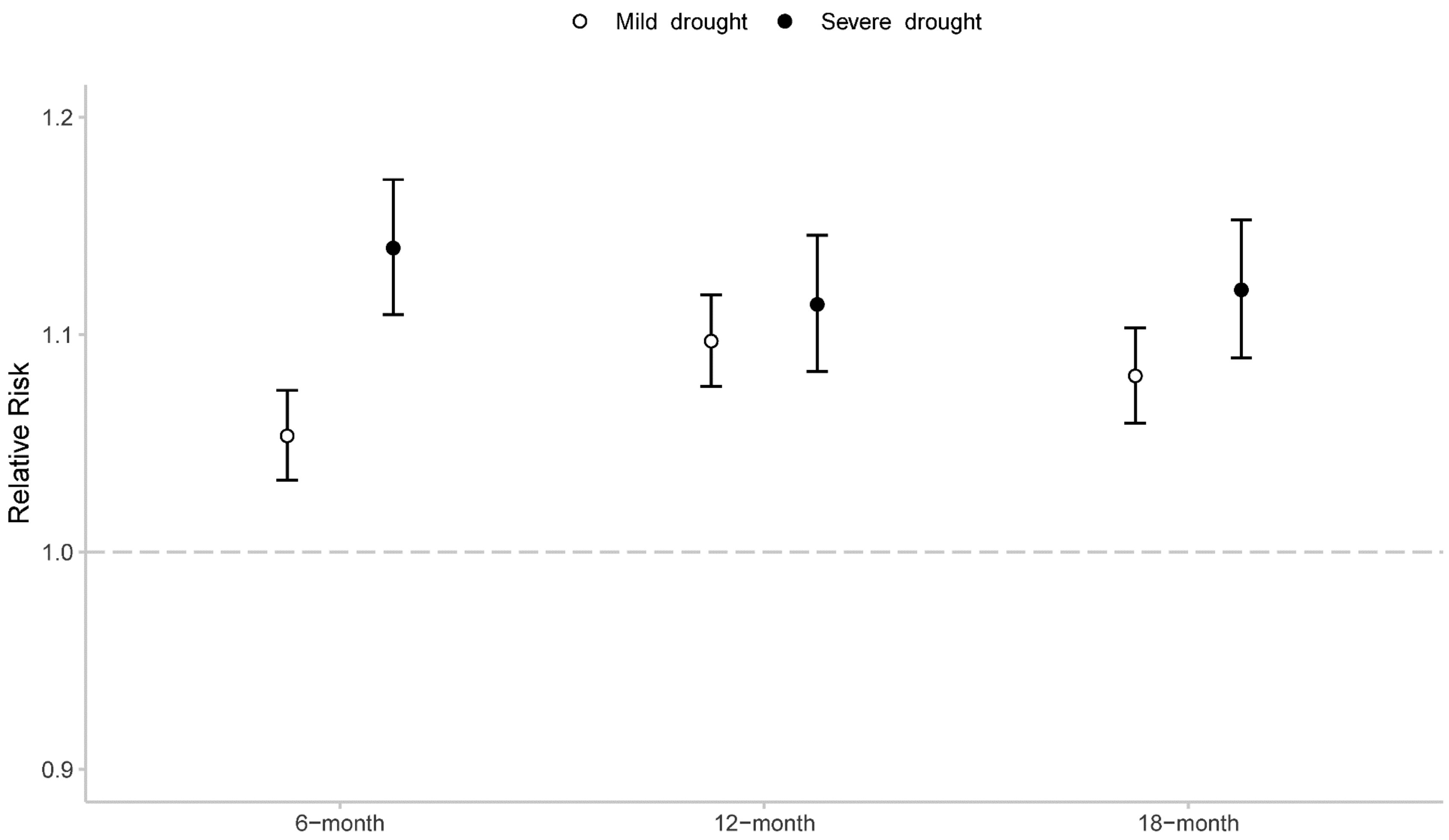

Supplementary Figure 17. Associations between diarrhea in children and drought at different timescales after multiple imputation (N=1,379,566). Generalized linear mixed effect models were used and no adjustments were made for multiple comparisons. Data are presented as mean values +/- 1.96 × standard error.

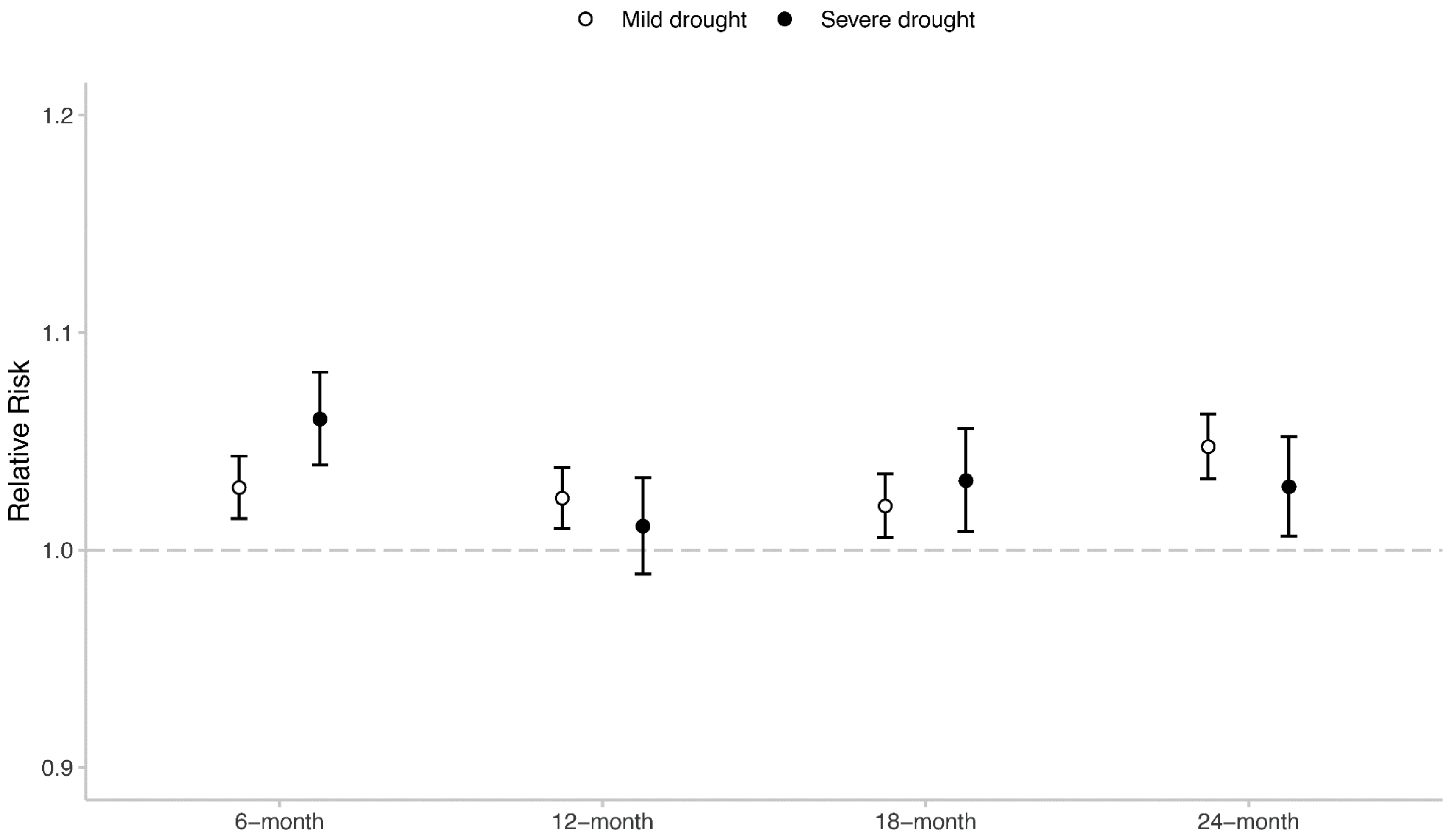

Supplementary Figure 18. Associations between diarrhea in children and drought at different timescales after disaggregating the drought variable (*N*=713,956). Generalized linear mixed effect models were used and no adjustments were made for multiple comparisons. Data are presented as mean values +/- 1.96 × standard error.

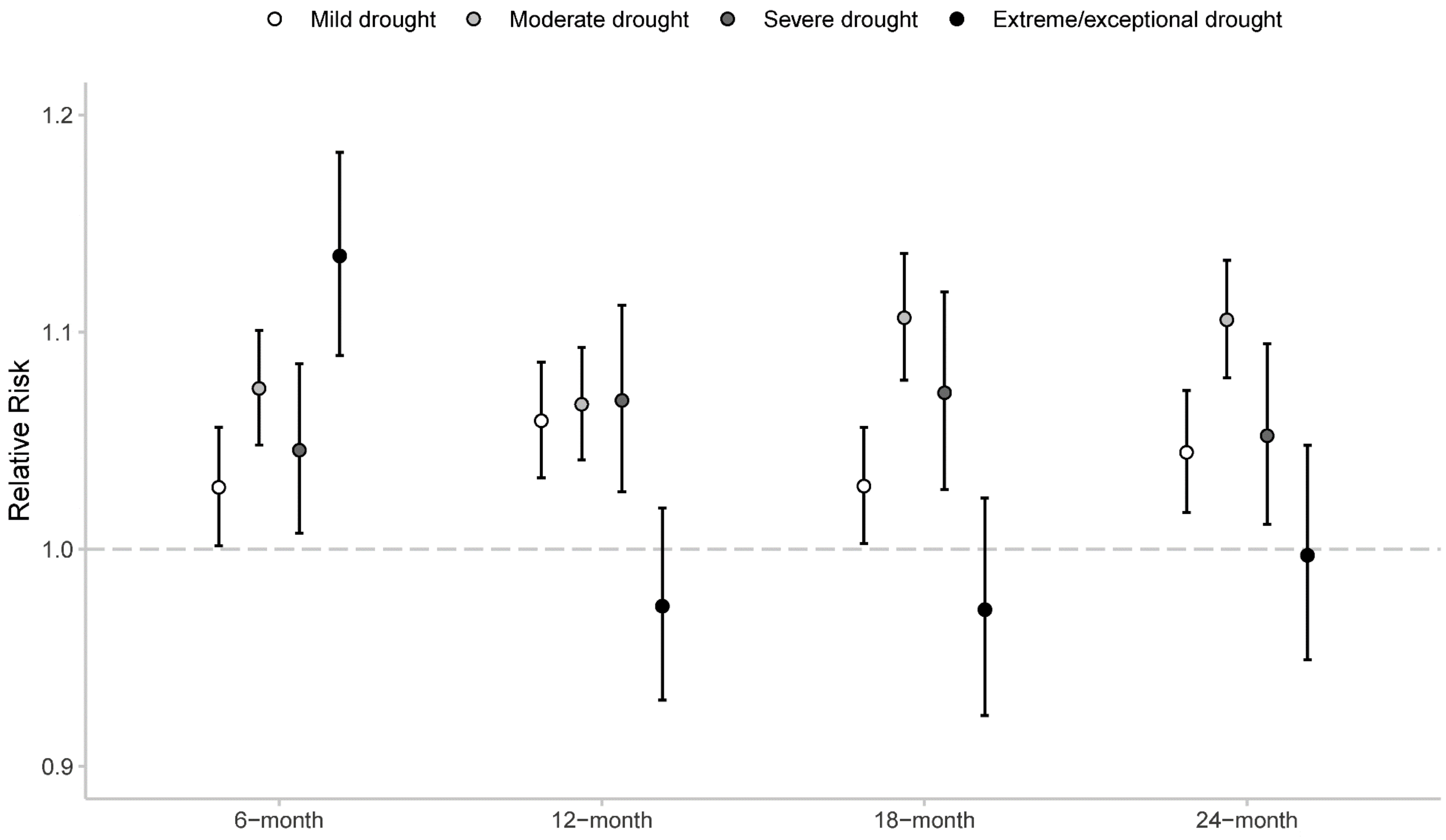

Supplementary Figure 19. Associations between diarrhea in children and drought at different timescales after including nutrition indicators as covariates (*N*=560,768). Generalized linear mixed effect models were used and no adjustments were made for multiple comparisons. Data are presented as mean values +/- 1.96 × standard error.

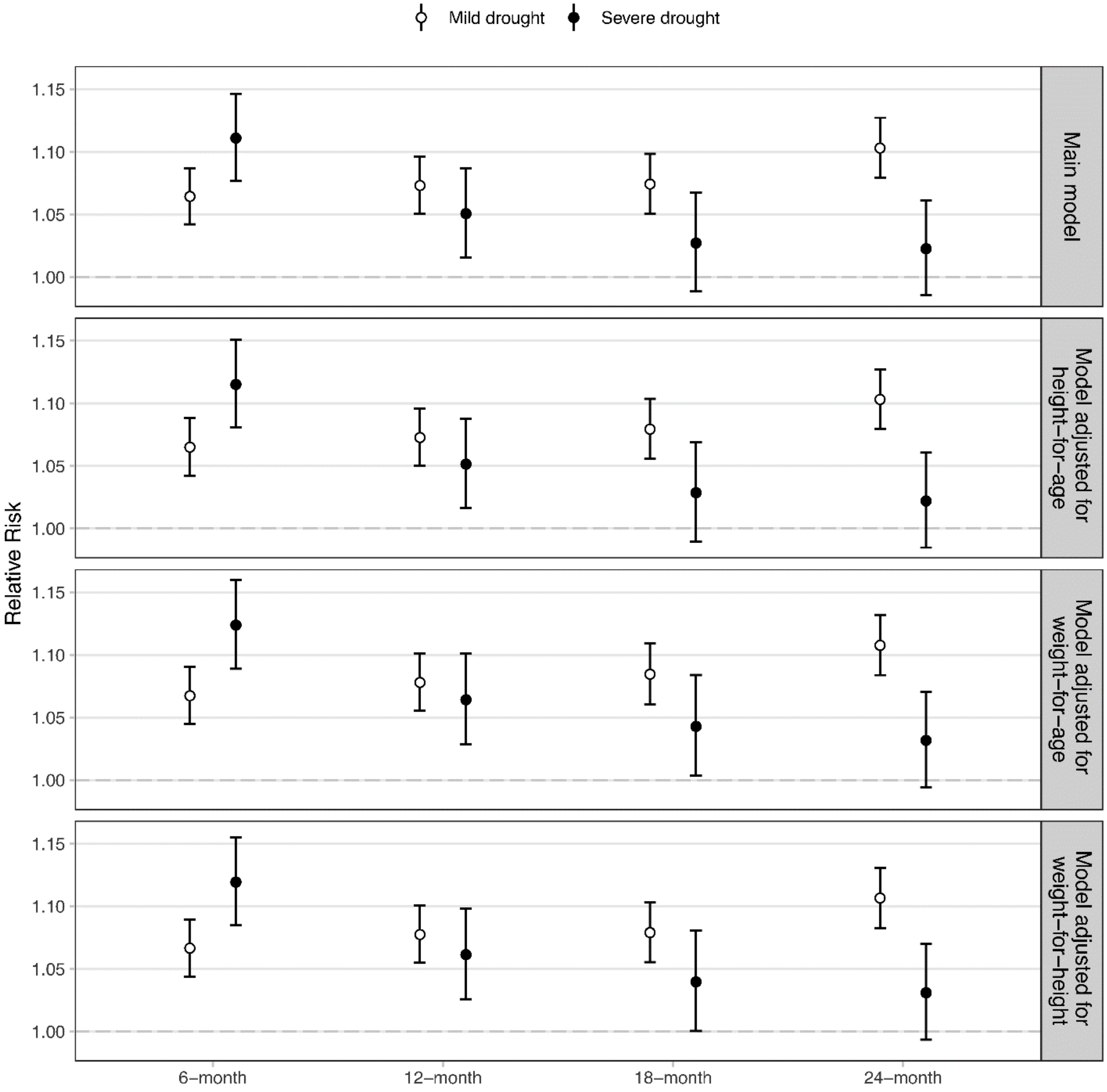

Reference:

1. VanderWeele TJ. A unification of mediation and interaction: a 4-way decomposition. *Epidemiology* **25**, 749-761 (2014).
