# Supplementary material for: Associations between long-term drought and diarrhea among children under five in low- and middle-income countries
Source: Nat Commun. 2022 Jun 30;13:3661. doi: 10.1038/s41467-022-31291-7 (PMC9247069; doi:10.1038/s41467-022-31291-7)
Supplement: Supplementary file 2 — Reporting Summary [file 41467_2022_31291_MOESM2_ESM.pdf]

nature portfolio

Corresponding author(s): Pin Wang

Last updated by author(s): May 10, 2022

Reporting Summary

Nature Portfolio wishes to improve the reproducibility of the work that we publish. This form provides structure for consistency and transparency in reporting. For further information on Nature Portfolio policies, see our Editorial Policies and the Editorial Policy Checklist.

Statistics

For all statistical analyses, confirm that the following items are present in the figure legend, table legend, main text, or Methods section.

n/a	Confirmed	
☐	☒	The exact sample size (n) for each experimental group/condition, given as a discrete number and unit of measurement
☒	☐	A statement on whether measurements were taken from distinct samples or whether the same sample was measured repeatedly
☐	☒	The statistical test(s) used AND whether they are one- or two-sided<br>*Only common tests should be described solely by name; describe more complex techniques in the Methods section.*
☐	☒	A description of all covariates tested
☐	☒	A description of any assumptions or corrections, such as tests of normality and adjustment for multiple comparisons
☐	☒	A full description of the statistical parameters including central tendency (e.g. means) or other basic estimates (e.g. regression coefficient) AND variation (e.g. standard deviation) or associated estimates of uncertainty (e.g. confidence intervals)
☐	☒	For null hypothesis testing, the test statistic (e.g. F, t, r) with confidence intervals, effect sizes, degrees of freedom and P value noted<br>*Give P values as exact values whenever suitable.*
☒	☐	For Bayesian analysis, information on the choice of priors and Markov chain Monte Carlo settings
☐	☒	For hierarchical and complex designs, identification of the appropriate level for tests and full reporting of outcomes
☒	☐	Estimates of effect sizes (e.g. Cohen's d, Pearson's r), indicating how they were calculated

Our web collection on statistics for biologists contains articles on many of the points above.

Software and code

Policy information about availability of computer code

Data collection	No software was used to collect the data.
Data analysis	All data analyses were completed using R statistical software (version 4.0.2), with the SPEI package (version 1.7) for the drought exposure assessment, and MASS package (version 7.3-54) for regression analysis. The programing code for the main models and mediation analysis is available at https://github.com/CHENlab-Yale/drought-diarrhea or https://doi.org/10.5281/zenodo.6527455.

For manuscripts utilizing custom algorithms or software that are central to the research but not yet described in published literature, software must be made available to editors and reviewers. We strongly encourage code deposition in a community repository (e.g. GitHub). See the Nature Portfolio guidelines for submitting code & software for further information.

Data

Policy information about availability of data

All manuscripts must include a data availability statement. This statement should provide the following information, where applicable:

- Accession codes, unique identifiers, or web links for publicly available datasets
- A description of any restrictions on data availability
- For clinical datasets or third party data, please ensure that the statement adheres to our policy

Survey data including diarrhea and socioeconomic data in this study are publicly available upon request from the Demographic and Health Surveys program (https://dhsprogram.com/). Publicly available meteorological records can be downloaded from the fifth generation ECMWF atmospheric reanalysis of the global climate (ERA5-Land) at https://cds.climate.copernicus.eu/. The SPEI at a resolution of 0.5° (~50×50 km) calculated by the Global SPEI database can be downloaded at https://spei.csic.es/spei_database. The SPEI at a resolution of 0.25° (~25×25 km) calculated by the European Space Agency can be downloaded at https://explorer-eo4sdcr.adamplatform.eu/. Source data on the calculated SPEI for the regression models and data on drought-month by drought severity and timescale are

provided with this paper at https://github.com/CHENlab-Yale/drought-diarrhea.

Field-specific reporting

Please select the one below that is the best fit for your research. If you are not sure, read the appropriate sections before making your selection.

- [x] Life sciences
- [ ] Behavioural & social sciences
- [ ] Ecological, evolutionary & environmental sciences

For a reference copy of the document with all sections, see nature.com/documents/nr-reporting-summary-flat.pdf

Life sciences study design

All studies must disclose on these points even when the disclosure is negative.

Sample size	We downloaded survey data from 51 low- and middle-income countries in sub-Saharan Africa, South and Southeast Asia, and Latin America and the Caribbean. In total we included 1,379,566 children reported by the mother, of which 713,918 were included in our main models due to missing values. We used the regression to estimate an overall association between drought exposure and diarrhea in children. This final sample size covers 43 countries and 30 years, which is statistically sufficient. In our preliminary power calculation, this sample size corresponded to a study power of 0.83-1.00, based on different risk estimates and ratio of exposed to unexposed population.
Data exclusions	We did not excluded any children in our study design. However, our regression analysis automatically excluded 665,648 observations due to missing values for baseline characteristics (primarily age and wealth index).
Replication	This study used survey data for diarrhea incidence in children and local meteorological data. Our findings are solely generated by the statistical analyses described in the methodology. All data required for the analyses are publicly available. And the programming code is available upon request from the authors. The authors have run the exposure assessment and regression analysis multiple times and identical results were observed.
Randomization	There was no experimental design or group allocation involved in this study.
Blinding	There was no experimental design or group allocation involved in this study.

Reporting for specific materials, systems and methods

We require information from authors about some types of materials, experimental systems and methods used in many studies. Here, indicate whether each material, system or method listed is relevant to your study. If you are not sure if a list item applies to your research, read the appropriate section before selecting a response.

Materials & experimental systems

n/a	Involved in the study	
[x]	[ ]	Antibodies
[x]	[ ]	Eukaryotic cell lines
[x]	[ ]	Palaeontology and archaeology
[x]	[ ]	Animals and other organisms
[ ]	[x]	Human research participants
[x]	[ ]	Clinical data
[x]	[ ]	Dual use research of concern

Methods

n/a	Involved in the study	
[x]	[ ]	ChIP-seq
[x]	[ ]	Flow cytometry
[x]	[ ]	MRI-based neuroimaging

Human research participants

Policy information about studies involving human research participants

Population characteristics	We included all children under five years old with diarrhea in the previous two weeks reported by the mother in 51 low- and middle-income countries, together with their sex and age (in month), mother's education, and rural/urban area of residence. Specifically, among these 1,379,566 children, 700,568 were males, 448,541 were younger than 24 months old, 408,245 lived in urban area, 940,153 children's mothers did not receive secondary or higher education.
Recruitment	This study did not recruit any children. Instead, we used secondary data surveyed by the Demographic and Health Surveys program.
Ethics oversight	This study was determined by the Yale Institutional Review Board as not-human-subject research (IRB ID: 2000030174) and thus ethics approval was not required for the current study.

Note that full information on the approval of the study protocol must also be provided in the manuscript.
